# Early Versus Late Dialysis in Cirrhosis Patients and Septic Shock (ELDICS Study): A Randomized Controlled Trial (NCT02937961)

**DOI:** 10.1002/jgh3.70216

**Published:** 2025-09-24

**Authors:** Rakhi Maiwall, Samba Siva Rao Pasupuleti, Prashant Agarwal, Sherin Thomas, Harsh Vardhan Tevethia, Rajendra Prasad Mathur, Shiv Kumar Sarin

**Affiliations:** ^1^ Department of Hepatology Institute of Liver and Biliary Sciences New Delhi India; ^2^ Department of Statistics, Mizoram University (A Central University) Pachhunga University, College Campus Aizawl India; ^3^ Department of Critical Care Institute of Liver and Biliary Sciences New Delhi India; ^4^ Department of Biochemistry Institute of Liver and Biliary Sciences New Delhi India; ^5^ Department of Nephrology Institute of Liver and Biliary Sciences New Delhi India

**Keywords:** ACLF, CKD, CRRT, cystatin C, NGAL, sepsis

## Abstract

**Background and Aim:**

Critically ill cirrhotics (CIC) pose a management challenge due to severe metabolic and renal impairment. The ideal timing of initiation of dialysis in acute kidney injury (AKI) in CIC is not known. We aimed to compare the safety and efficacy of early (ED) versus late (LD) initiation of sustained low‐efficiency dialysis (SLED) in CIC.

**Methods:**

CIC were randomized to ED (SLED initiated within 6–12 h) or the LD (where SLED was performed when the patient met absolute criteria) group.

**Results:**

Fifty CIC (aged 45.2 ± 10 years), 90% males, 87% alcohol‐related, 72% with pneumonia admitted to liver ICU were randomized to ED or LD group. Baseline lactate (mg/dL) (2.7 ± 1.8 vs. 3.3 ± 2.1) and SOFA scores (12.9 ± 2.1 vs. 13.7 ± 4.0) were comparable. Median time to dialysis (in hours) was 7 (IQR 6–8) in ED and 24 (18–48) in LD group. Mortality at 28 days (56% vs. 76%; *p* = 0.14) was similar. A significantly lower incidence of intradialytic hypotension (IDH) (12% vs. 48%; *p* = 0.005), and better urea reduction (75% vs. 41%, *p* = 0.019), reversal of shock (60% vs. 16%; *p* = 0.001), renal functions (68% vs. 12%; *p* < 0.001), and lower early deaths at Day 7 were noted in the ED (20% vs. 52%; *p* = 0.038).

**Conclusions:**

Timely initiation of dialysis could avert the development or progression of metabolic complications, decrease the incidence of IDH and early deaths in CIC. A higher frequency of recovery of renal functions and reduced AKI‐related mortality could be achieved by timely dialysis in CICs.

**Trial Registration:** NCT02937961

**Abbreviations**
AKI: acute kidney injury
AKIKI: artificial kidney initiation in kidney injury
ATN: acute tubular necrosis
AUROC: area under the receiver operating characteristic curve
CI: confidence interval
CRRT: continuous renal replacement therapy
CysC: cystatin C
ED: early dialysis
HRS: hepatorenal syndrome
IDH: intradialytic hypotension
INR: international normalized ratio
IQR: interquartile range
KDIGO: kidney disease improving global outcome criteria
LD: late dialysis
L‐ICU: liver intensive care unit
NGAL: neutrophil gelatinase associated lipocalin
NT: pro‐BNP‐N‐terminal pro‐brain natriuretic peptide MELD‐model for end‐stage liver disease
OR: odds ratio
RRT: renal replacement therapy
SLED: sustained low‐efficiency dialysis
SNOSE: sequentially numbered opaque sealed envelopes
SOFA: sequential organ failure assessment
TLC: total leukocyte count

## 1 Introduction

Acute kidney injury (AKI) is seen in 80% of the critically ill patients with cirrhosis admitted to the liver intensive care unit (L‐ICU) [1]. Almost 50% of the episodes of AKI are severe and constituted by acute tubular necrosis (ATN) [2]. The management of AKI in patients with cirrhosis admitted to the intensive care unit is challenging [2]. The mortality of patients with AKI and multiorgan failure varies from 50% to 80% [3, 4]. In the absence of any standard guidelines for managing AKI in the context of critically ill patients with cirrhosis, the control of electrolyte abnormalities, acid–base balance, and volume control by using renal replacement therapy (RRT) remains the only effective option for management [4].

There are several unanswered questions regarding the exact timing, modality, and patient selection for RRT in critically ill individuals. These questions are quintessential for improving overall patient outcomes with AKI [5]. In patients with absolute indications for RRT, the initiation is performed as soon as possible, as any delay is associated with imminent death. However, in patients with relative criteria, a timely initiation once the patient is in the early phase of organ dysfunction, rather than late, may improve renal and nonrenal outcomes [6, 7]. However, the evidence supporting this notion is sparse and has not been supported by randomized controlled trials [6, 7, 8]. Moreover, patients with cirrhosis and those with hepatorenal syndrome were mostly excluded from these trials. There is currently a paucity of studies investigating the initiation of RRT in patients with cirrhosis with sepsis and multiorgan failure.

Sustained‐low efficiency dialysis (SLED) is a hybrid modality of dialysis which is very popular in most intensive care units [9, 10]. It has the advantage of better solute and metabolic control, hemodynamic stability, better efficiency at a reduced cost, and could be the ideal modality of dialysis in resource‐constrained countries. Randomized controlled trials performed in the context of critically ill have failed to show a superiority of continuous RRT over intermittent modalities of dialysis [10]. We hypothesized that cirrhotics with septic shock have limited organ reserve and poor outcomes with dialysis timed late; a preemptive initiation of SLED in critically ill patients with septic shock may be superior in improving overall outcomes by averting the metabolic complications.

## 2 Methods

### 2.1 Study Design

The study was designed as an open‐label randomized controlled trial conducted at the Institute of Liver and Biliary Sciences, New Delhi, India from January 2018 to September 2019. The protocol was approved by the appropriate legal authority and is summarized in the Supporting Information Appendix. The study protocol was registered with ClinicalTrial.gov (identifier: NCT02937961). All authors had access to the study data and reviewed and approved the final manuscript.

### 2.2 Participants

We screened and enrolled consecutive patients with cirrhosis with septic shock and AKI admitted to the L‐ICU. Patients with Kidney Disease Global Outcome criteria (KDIGO) Stage 2 or more after appropriate fluid resuscitation were included and randomized. We excluded patients aged less than 18 years, with severe known cardiopulmonary disease, pregnant patients, patients with chronic kidney disease, postrenal obstructive AKI, patients with hepatorenal syndrome (HRS‐AKI), patients who already met absolute criteria for dialysis as specified in the late group, patients transferred in from other hospitals or wards who had already received the hemodialysis, extremely moribund patients with an expected life expectancy less than 24 h, or those with refractory shock and lack of informed consent.

### 2.3 Randomization and Masking

Randomization was done by the clinical trial coordinator using the block randomization method with a block size of 10 with 5 blocks. Allocation concealment was performed by the SNOSE technique (sequentially numbered opaque sealed envelopes) provided by the trial coordinator.

#### 2.3.1 Interventions

In the early strategy group (ED), SLED was initiated within 6–12 h of randomization, while in the delayed‐strategy group (LD) SLED was performed only once the patient met absolute criteria for dialysis. These included severe metabolic acidosis, hyperkalemia refractory to medical management, volume overload nonresponsive to intravenous diuretics, or progressive worsening azotemia (details in Supporting Information Appendix).

#### 2.3.2 Outcome Measures

The primary outcome was deaths due to any cause at 28 days. The secondary outcomes included assessment of hemodynamic stability, recovery of shock and renal functions, improvement in sequential organ failure assessment (SOFA) score, duration of mechanical ventilation, and intensive care unit stay. We also aimed to study the role of renal biomarkers, that is, urine neutrophil‐gelatinase associated lipocalin (NGAL), cystatin C (CysC), and N‐terminal pro‐brain natriuretic peptide (NT‐pro‐BNP) in predicting clinical outcomes. Renal recovery was defined as discontinuation of maintenance dialysis with an increase in urine output to more than 400 mL/day in patients who were anuric [2, 3].

## 3 Statistical Methods

### 3.1 Sample‐Size

Considering the lack of data on RRT in patients with cirrhosis, the study was designed as a pilot trial with an aim to perform an interim analysis after 3 months for calculation of sample size. The interim analysis demonstrated that most of the deaths secondary to the intervention were early. Multiple other factors contributed to 28‐day survival in such patients; therefore, the protocol was revised to include early deaths at 7 days, and because the data from the interim analysis suggested a clear harm in the delayed strategy, it was decided to terminate the trial after enrolment of 50 patients, as suggested by the institutional ethics committee. The results of the interim analysis were presented as an abstract.

### 3.2 Statistical Analysis

Continuous variables were compared between the ED and LD groups using independent samples Student's *t* test or Mann–Whitney *U* test for normal or nonnormally distributed data, respectively. Categorical variables were analyzed using Fischer's exact test or the chi‐square test between the two randomized groups of patients. Intention to treat analysis and per‐protocol (PP) analyses were performed. Cox regression was performed for survival analysis. All tests were two‐tailed, and *p* < 0.05 was considered significant. Multivariable binary statistical analysis was done by using the statistical package for social sciences (IBM corp Ltd. Armonk, NY version 22.0).

## 4 Results

**FIGURE 1:**
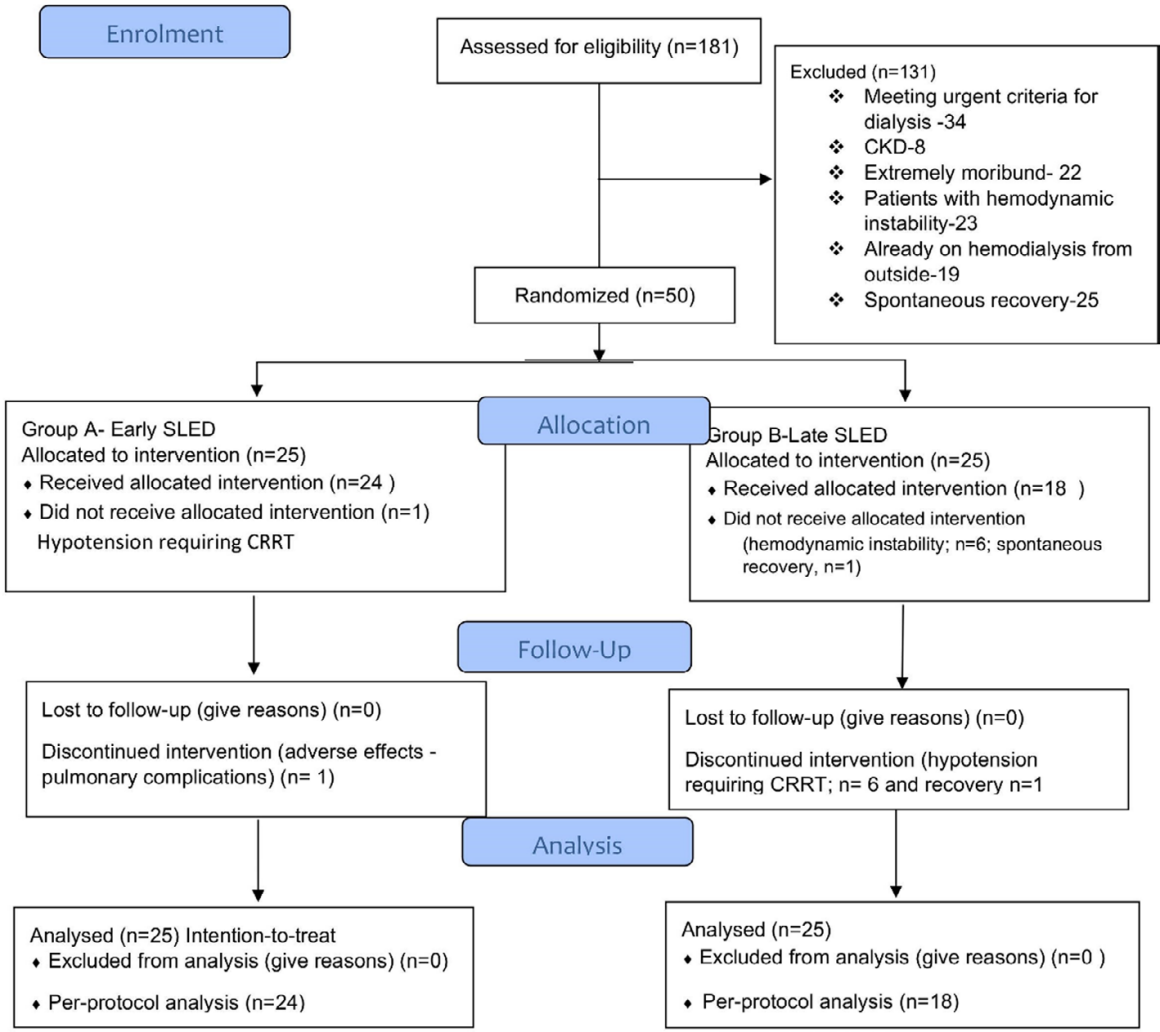
Consort flow diagram for patients enrolled in the trial.

From January 2018 to September 2019, a total of 181 patients with septic shock and KDIGO 2 or higher were screened for randomization. However, the final number of patients included was 50, with 25 in the ED group and 25 in the LD group (Figure 1). The mean age of the included patients was 45.2 ± 10.0 years; 90% were males, and in the majority the predominant etiology was alcohol (87%). Baseline demographic and renal parameters were comparable. Median time to dialysis (in hours) was 7 (interquartile range; IQR 6–8) in ED vs. 24 (18–48) h in LD. (Table 1).

**TABLE 1:** Baseline characteristics of the study cohort (*n* = 50).

	Early group (*n* = 25)	Late group (*n* = 25)	*p*
Age (in years)	46.12 ± 9.64	44.32 ± 10.39	0.53
Gender (males); *n* (%)	23 (92)	22 (88)	1.00
Etiology (alcohol); *n* (%)	14 (56%)	16 (64%)	0.56
Severity scores			
Model for end‐stage liver disease	34.76 ± 6.05	34.72 ± 6.95	0.98
Sequential organ failure assessment	12.92 ± 2.14	13.68 ± 3.98	0.40
Child turcott pugh score	12.24 ± 1.54	12.68 ± 1.45	0.34
Physiological parameters			
Mean arterial pressure (mm of Hg)	73.88 ± 10.40	78.32 ± 19.23	0.31
Heart rate (beats/min)	95.84 ± 16.89	99.44 ± 22.84	0.53
Respiratory rate (/min)	20.32 ± 3.04	20.64 ± 3.77	0.74
Urine Output (ml/h)	18.00 ± 11.90	20.80 ± 21.20	0.57
KDIGO; *n* (%)			
Stage 3	15 (60)	17 (68)	0.77
Stage 2	10 (40)	8 (32)	
FiO_2_ (%)	49.36 ± 25.66	49.06 ± 24.24	0.97
Arterial lactate (mmol/L)	2.73 ± 1.82	3.26 ± 2.13	0.35
Central venous pressure (in cm)	12.68 ± 2.39	12.6 ± 2.33	0.91
IVC (diameter in cm)	19.08 ± 3.26	18.99 ± 3.38	0.92
pH	7.36 ± 0.12	7.36 ± 0.11	0.83
pCO_2_ (mm of Hg)	35.8 ± 9.11	36.62 ± 17.77	0.84
pO_2 (_mm of Hg)	113.01 ± 47.68	108.96 ± 32.92	0.73
Biochemical parameters			
Total leucocyte count (× 10^3^ cells/mm^3^)	11.26 ± 6.73	14.72 ± 8.96	0.13
Platelet count (× 10^3^ cells/mm^3^)	113.44 ± 63.88	133.95 ± 62.84	0.26
Serum total bilirubin (mg/dL)	13.91 ± 11.51	10.23 ± 8.54	0.21
International normalized ratio	1.94 ± 0.63	2.14 ± 1.05	0.43
Hemoglobin (g/dL)	9.77 ± 2.66	9.51 ± 1.67	0.69
Serum sodium (mEq/L)	136.66 ± 7.95	132.06 ± 7.95	0.046
Serum potassium (mEq/L)	4.19 ± 0.73	4.33 ± 0.69	0.47
Serum bicarbonate (mEq/L)	20.68 ± 4.48	18.97 ± 3.84	0.16
Anion gap	7.74 ± 4.04	6.29 ± 7.36	0.39
Serum calcium (mg/dL)	5.68 ± 3.53	4.21 ± 3.75	0.16
Serum magnesium (mg/dL)	2.58 ± 0.98	2.33 ± 0.41	0.24
Serum phosphate (mg/dL)	3.47 ± 1.28	3.92 ± 1.78	0.31
Serum creatinine (mg/dL)	3.00 ± 1.49	2.66 ± 0.60	0.30
Serum urea (mg/dL)	94.71 ± 54.79	85.65 ± 72.01	0.64
Serum chloride (mEq/L)	110.54 ± 5.14	105.25 ± 6.03	0.002
Vasopressors			
Norepinephrine dose (μg/min)	4.24 ± 2.55	3.44 ± 2.18	0.24
Vasopressin (mL/h)	0.70 ± 0.92	0.56 ± 1.01	0.62
Biomarkers			
Urine neutrophil gelatinase lipocalin (ng/mL)	2091.37 ± 1928.03	2359.79 ± 1738.49	0.61
Serum cystatin C (mg/L)	2.71 ± 0.63	3.04 ± 0.71	0.09
NT‐pro‐BNP (pg/mL)	2632.00 ± 2036.25	4677.33 ± 3395.89	0.035
Site of infection; *n* (%)			
Pneumonia	17 (68)	19 (76)	0.17
Spontaneous bacterial peritonitis	0 (0)	2 (8)	
Spontaneous bacteremia	5 (20)	1 (4)	
Urinary tract infection	3 (12)	2 (8)	
Others	0 (0)	1 (4)	
Organism; *n* (%)	5 (20)	0 (0)	0.055
Bacteria			
Gram‐negative			
Acinetobacter baumanii	5 (20)	0 (0)	
Klebsiella pneumoniae	7 (28)	9 (36)	
Pseudomonas	2 (8)	4 (24)	
Escherichia coli	2 (8)	1 (4)	
Stenotrophomonas	1 (4)	0 (0)	
Gram‐positive			
Vancomycin‐resistant enterococci (VRE)	1 (4)	3 (12)	
Methicillin‐resistant *Staphylococcus aureus*	0 (0)	2 (8)	
Fungi			
Candida	5 (20)	3 (12)	
Aspergillus	2 (8)	1 (4)	
Indication of dialysis; *n* (%)			
Azotemia	19 (76)	18 (72)	0.75
Hyperkalemia	6 (24)	6 (24)	1.00
Metabolic acidosis	18 (72)	15 (60)	0.37
Uremic encephalopathy	6 (24)	2 (8)	0.25
Uremic gastritis	2 (8)	1 (4)	1.00
Fluid overload	6 (24)	7 (28)	0.75
Hyperlactetemia	17 (68)	10 (40)	0.01
Hyponatremia	5 (20)	6 (24)	0.73
Hypernatremia	7 (28)	0 (0)	0.004
Ascites; *n* (%)			
Grade 1	1 (4)	2 (8)	0.38
Grade 2	23 (92)	19 (76)	
Grade 3	1 (4)	4 (16)	
Hepatic encephalopathy; *n* (%)			
Grade 0	2 (8)	0 (0)	0.49
Grade 1 or 2	1 (4)	3 (12)	
Grade 3 or 4	1 (4)	1 (4)	

*Note:* Plus–minus values are means ± SD. Categorical variables are depicted as number (percentage). *p* Values were given for the comparison between the group of patients in early dialysis versus the late dialysis groups. *p* Values for categorical variables were calculated with the use of Chi‐square test. *p* Values for continuous variables were calculated with the use of unpaired Student's *t* test.

Abbreviations: FiO_2_, fraction of inspired oxygen; IVC, inferior vena cava diameter; KDIGO, kidney disease improving global outcome criteria; PCO_2_, partial pressure of carbon dioxide; PO_2_, partial pressure of oxygen; SOFA, sequential organ failure assessment.

### 4.1 Intention‐To‐Treat Analysis (Table S1)

#### 4.1.1 Primary Outcome

**FIGURE 2:**
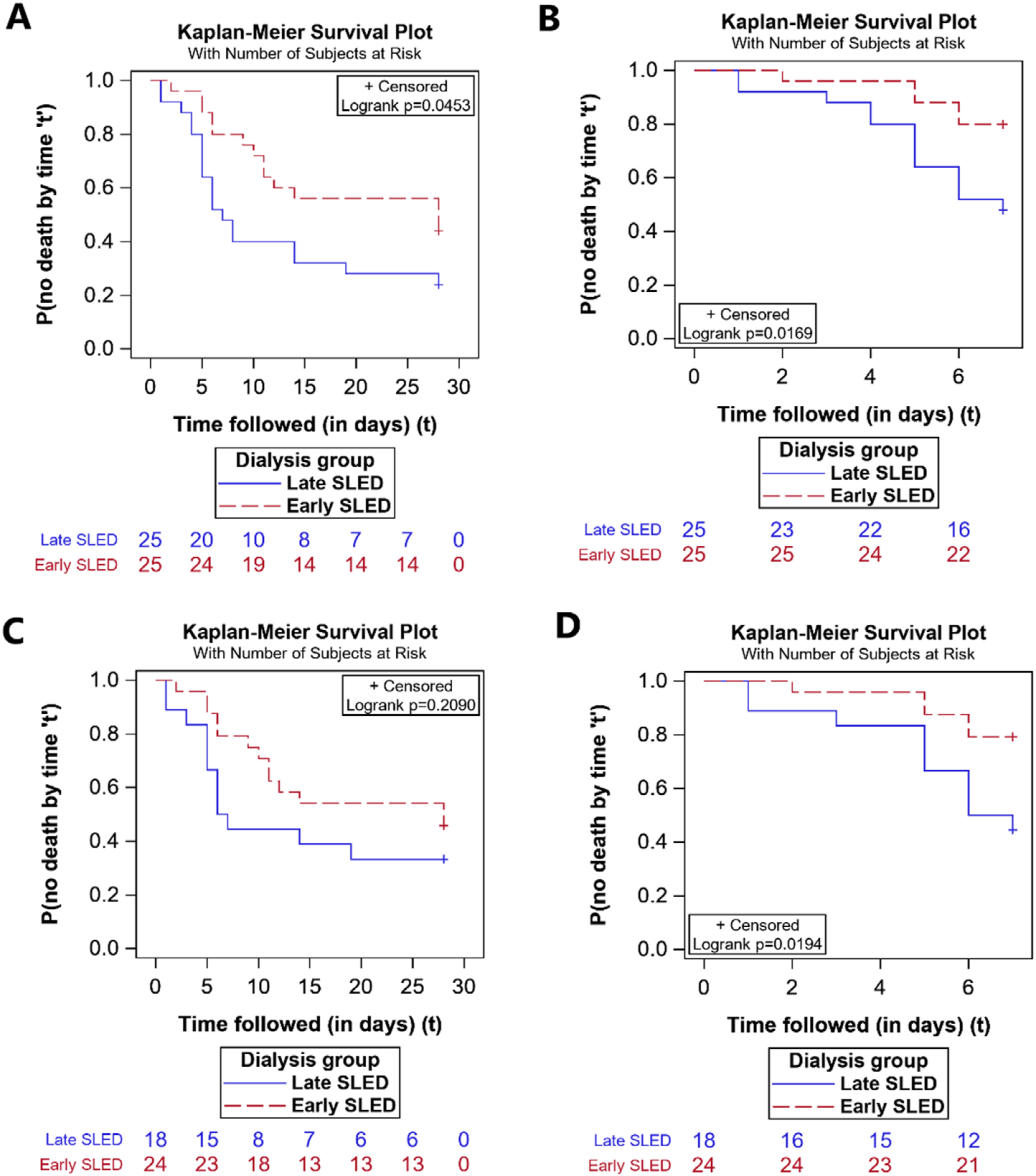
Kaplan–Meier survival analysis stratified by early versus late initiation of dialysis. For 28‐ and 7‐days mortality. (A) Patients stratified by the dialysis strategy for 28‐day mortality on intention‐to‐treat analysis. (B) Patients stratified by the dialysis strategy for 7‐day mortality on intention‐to‐treat analysis. (C) Patients stratified by the dialysis strategy for 28‐day mortality on per‐protocol analysis. (D) Patients stratified by the dialysis strategy for 7‐day mortality on per‐protocol analysis.

The mortality at 28 days was not different between the two groups ED versus LD (14 [56%] vs. 19 [76%]; *p* = 0.14) (Figure 2A). However, the early deaths (i.e., deaths within the first 7 days of randomization) were significantly higher in the LD (52% vs. 20%; *p* = 0.038) (Figure 2B). Of these deaths, 36% (10 out of 28) patients had developed intradialytic hypotension (IDH). The majority of these patients were in the late group (69% [9 out of 13] vs. 20% [1 out of 5]). Of these patients, three in the LD versus none in the ED had protocol violations and required CRRT.

#### 4.1.2 Secondary Outcomes

##### 4.1.2.1 Incidence of IDH

Patients in LD developed a higher incidence of IDH and hemodynamic instability compared to ED (12 [48%] vs. 3 [12%]; *p* = 0.005) and (19 [76%] vs. 14 [56%]; *p* = 0.14). The development of IDH was associated with discontinuation of dialysis in both groups.

#### 4.1.3 Urea Reduction Ratio and Achievement of Target Ultrafiltration Goals

A significantly higher urea reduction and achievement of target ultrafiltration was observed in ED vs. LD (18 [75%] vs. 9 [41%]; *p* = 0.019) and (15 [65%] vs. 6 [33%]; *p* = 0.04).

#### 4.1.4 Duration of Mechanical Ventilation and ICU Stay

The duration of mechanical ventilation (3.92 ± 3.32 vs. 5.56 ± 4.39; *p* = 0.14] and ICU stay ([5.67 ± 3.28] vs. [6.28 ± 4.52]; *p* = 0.59) was not different between the ED and LD, respectively.

#### 4.1.5 Reversal of Shock and Impact on Arterial Lactate

A significantly higher proportion of patients achieved reversal of shock in the ED compared to LD (15 [60%] vs. 4 [16%]; *p* = 0.001). There was no difference in the arterial lactate at 0, 6,12, 24, and 48 h between the two groups.

### 4.2 PP Analysis

Almost similar results were observed in PP (Table S1). The early deaths at Day 7 were significantly higher in the LD compared to ED (56% [10 out of 18] vs. 21% [5 out of 24]; *p* = 0.027) (Figure 2C,D).

### 4.3 Renal Biomarkers

Renal biomarkers did not predict 28‐day mortality; however, a lower CysC predicted renal recovery (odds ratio [OR] 0.18, 95% CI 0.05–0.66) while uNGAL predicted reversal of shock (OR 0.61, 95% CI [0.39–0.95]). A CysC value of ≤ 2.75 better predicted renal recovery with a sensitivity of 70.0% and specificity of 66.6% AUROC 0.698, PPV 58.33%, NPV 76.92%, OR 4.66, 95% CI [1.376–15.823]) while a CysC value of ≥ 3.53 better predicted 7‐day mortality with a sensitivity of 55.6% and specificity of 84.4% (AUROC 0.709, PPV 66.67%, NPV 77.14%, OR 6.75, 95% CI (1.781–25.580).

### 4.4 Predictors of Renal Recovery

The recovery of renal functions was higher in patients with ED compared to LD (17 [68%] vs. 3 [12%]; *p* < 0.001). The time taken for renal recovery (in hours) was, however, not significantly different between the two groups ED versus LD (2.65 ± 1.65 vs. 1.33 ± 1.15; *p* = 0.21). On multivariate analysis, in Model‐1, ED OR 13.7 (1.87–100.29) and higher platelet count OR 1.02 (1.00–1.03) and in Model‐2, wherein we excluded the intervention group, a lower CysC OR 0.18 (0.04–0.77), higher platelet counts OR 1.02 (1.00–1.03) and absence of IDH OR 0.10 (0.01–0.91) were independent predictors of renal recovery (Table 2). Similar factors were identified as independent predictors on the PP analysis (Table S2).

**TABLE 2:** Predictors of renal recovery—Logistic regression analysis.

Variables	Model 1 (unadjusted odds ratio)		Model 2		Model 3	
	*p*	OR (95% CI)	*p*	OR (95% CI)	*p*	OR (95% CI)
Age (in years)	0.37	1.03 (0.97–1.09)				
Gender (males); *n* (%)	0.35	0.40 (0.06, 2.67)				
Etiology (alcohol); *n* (%)	0.556	1.42 (0.44–4.57)				
Severity scores						
Model for end‐stage liver disease	0.60	0.98 (0.89–1.07)				
Sequential organ failure assessment	0.65	0.96 (0.80–1.15)				
Child‐Turcott‐Pughh score	0.74	0.93 (0.63–1.39)				
Physiological factors						
Mean arterial pressure (mm of Hg)	0.36	0.98 (0.94–1.02)				
Heart rate (beats/min)	0.76	1.005 (0.98–1.03)				
Respiratory rate (/min)	0.59	1.05 (0.89–1.24)				
Urine output (mL/h)	0.71	1.006 (0.97–1.04)				
KDIGO; *n* (%) Stages 3 vs. 2	0.63	0.75 (0.23–2.42)				
FiO_2_ (%)	0.67	1.005 (0.98–1.03)				
Arterial lactate (mmol/L)	0.13	0.77 (0.56–1.08)				
Central venous pressure (in cm)	0.44	1.10 (0.86–1.41)				
IVC (diameter in cm)	0.65	0.96 (0.81–1.14)				
pH	0.67	3.09 (0.16–581.41)				
pCO_2_ (mm of Hg)	0.62	0.99 (0.95–1.04)				
pO_2_ (mm of Hg)	0.35	0.99 (0.98–1.008)				
Biochemical parameters						
Total leucocyte count (× 10^3^ cells/mm^3^)	0.350	1.03 (0.96, 1.11)				
Platelet count (× 10^3^ cells/mm^3^)	0.006	1.02 (1.00, 1.03)	0.017	1.02 (1.00, 1.03)	0.013	1.02 (1.00, 1.03)
Serum total bilirubin (mg/dL)	0.260	0.97 (0.91, 1.03)				
International normalized ratio	0.547	0.80 (0.38, 1.67)				
Hemoglobin (g/dL)	0.367	0.88 (0.67, 1.16)				
Serum sodium (mEq/L)	0.71	0.99 (0.92–1.06)				
Serum potassium (mEq/L)	0.88	0.94 (0.42–2.09)				
Serum bicarbonate (mEq/L)	0.68	1.03 (0.89–1.18)				
Anion gap	0.55	1.03 (0.94–1.14)				
Serum calcium (mg/dL)	0.15	1.12 (0.96–1.32)				
Serum magnesium (mg/dL)	0.19	1.95 (0.71–5.35)				
Serum phosphate (mg/dL)	0.13	0.70 (0.45–1.11)				
Serum creatinine (mg/dL)	0.37	0.77 (0.44–1.36)				
Serum urea (mg/dL)	0.67	0.99 (0.99–1.008)				
Serum chloride (mEq/L)	0.16	1.08 (0.97–1.19)				
Vasopressor dose						
Norepinephrine (μg/min)	0.38	1.11 (0.88–1.41)				
Vasopressin (units/min)	0.84	1.06 (0.59–1.93)				
Biomarkers						
Urine neutrophil gelatinase lipocalin (ng/mL)	0.16	0.74 (0.49–1.13)				
Serum cystatin C (mg/L)	0.02	0.30 (0.11–0.83)	0.113	0.30 (0.07, 1.33)	0.021	0.18 (0.04, 0.77)
NT‐pro‐BNP	0.37	0.54 (0.14–2.11)				
Shock reversal	0.011	4.93 (1.44–16.88)				
Early dialysis (vs. late dialysis as ref.)	< 0.001	15.58 (3.58–67.78)	0.010	13.70 (1.87, 100.29)		
Intradialytic hypotension	0.02	0.15 (0.03–0.74)			0.041	0.10 (0.01, 0.91)
Lactate clearance at 24 h	0.03	4.00 (1.14–14.00)	0.546	1.85 (0.25, 13.48)	0.161	3.59 (0.60, 21.40)

*Note:* Data presented as odds ratio (OR) and 95% CI (confidence intervals) derived from binary logistic regression analysis.

Abbreviations: FiO_2_, fraction of inspired oxygen; IVC, inferior vena cava diameter; KDIGO, kidney disease improving global outcome criteria; PCO_2_, partial pressure of carbon dioxide; PO_2_, partial pressure of oxygen; SOFA, sequential organ failure assessment.

### 4.5 Predictors of 28‐Day Mortality

**FIGURE 3:**
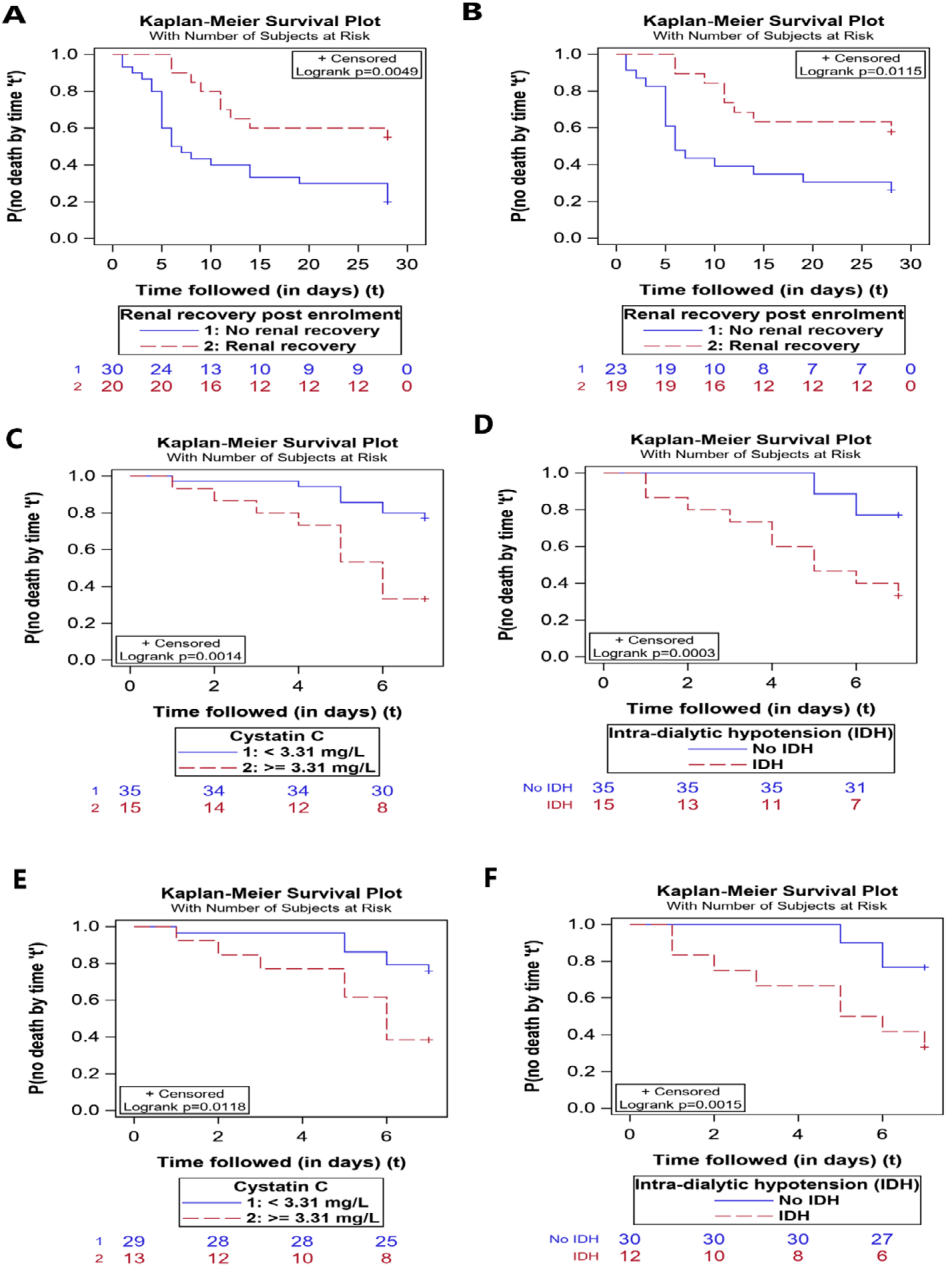
Kaplan–Meier survival curve stratified by patients who achieved renal recovery post enrolment versus those who did not irrespective of the randomization group. (A) The graph shows patients with renal recovery had improved 28‐day survival compared to no recovery on intention‐to‐treat analysis. (B) The graph shows patients with renal recovery had improved 28‐day survival compared to no recovery on per‐protocol analysis. (C) Patients stratified by cystatin C levels for 7‐day mortality on intention‐to‐treat analysis. (D) Patients stratified by presence or absence of intradialytic hypotension for 7‐day mortality on intention‐to‐treat analysis. (E) Patients stratified by cystatin C levels for 7‐day mortality on per‐protocol analysis. (F) Patients stratified by presence or absence of intradialytic hypotension for 7‐day mortality on per‐protocol analysis.

We created two multivariate models. In the first model, higher SOFA score was independently associated with worse outcomes. Renal recovery was associated with improved outcomes (*p* = 0.04, HR 0.42, 0.19–0.94) while it showed a trend toward significance in model 2 (*p* = 0.06, HR 0.42, 0.17–1.04) (Table 3, Figure 3A). On PP analysis, higher arterial lactate and lack of renal recovery were associated with higher deaths at 28 days (Table S3, Figure 3B). Patients with renal recovery also had lower 7‐day mortality both in the ITT and PP analysis (HR 0.14 [0.03–0.61] and 0.14 [0.03–0.61]) respectively.

**TABLE 3:** Predictors of 28‐day mortality—Cox‐regression (intention‐to‐treat) analysis.

Variables	Unadjusted		Model 1		Model 2	
	*p*	HR (95% CI)	*p*	HR (95% CI)	*p*	HR (95% CI)
Age (in years)	0.52	0.99 (0.96–1.02)				
Gender (males); *n* (%)	0.24	2.37 (0.57–9.90)				
Etiology (alcohol); *n* (%)	0.99	1.00 (0.50–2.01)				
Severity scores						
Model for end‐stage liver disease	0.51	1.02 (0.96–1.08)				
Sequential organ failure assessment	0.006	1.18 (1.05–1.33)	0.040	1.14 (1.01–1.29)	0.042	1.14 (1.00–1.29)
Child turcott pugh score	0.76	1.04 (0.82–1.32)				
Physiological parameters						
Mean arterial pressure (mm of Hg)	0.94	1.00 (0.98–1.03)				
Urine output (ml/h)	0.31	1.01 (0.99–1.04)				
KDIGO; *n* (%) Stages 3 vs. 2	0.50	1.28 (0.62–2.64)				
FiO_2_ (%)	0.73	1.00 (0.99–1.02)				
Arterial lactate (mmol/L)	0.02	1.19 (1.03–1.39)	0.190	1.11 (0.95–1.30)	0.195	1.11 (0.95–1.30)
Central venous pressure (in cm)	0.93	1.01 (0.87–1.16)				
IVC (diameter in cm)	0.31	1.06 (0.95–1.17)				
pH	0.82	0.68 (0.02–18.35)				
pCO_2_ (mm of Hg)	1.00	1.00 (0.97–1.03)				
pO_2_ (mm of Hg)	0.41	1.00 (0.99–1.01)				
Biochemical parameters						
Total leucocyte count (× 10^3^ cells/mm^3^)	0.670	1.01 (0.97, 1.05)				
Platelet count (× 10^3^ cells/mm^3^)	0.494	1.00 (0.99, 1.00)				
Serum total bilirubin (mg/dL)	0.529	1.01 (0.98, 1.04)				
International normalized ratio	0.641	1.08 (0.78, 1.49)				
Hemoglobin (g/dL)	0.368	1.08 (0.91, 1.28)				
Serum sodium (mEq/L)	0.73	1.01 (0.97–1.05)				
Serum potassium (mEq/L)	0.48	0.84 (0.51–1.37)				
Serum bicarbonate (mEq/L)	0.80	0.99 (0.91–1.07)				
Anion gap	0.39	1.03 (0.97–1.09)				
Serum calcium (mg/dL)	0.41	1.04 (0.94–1.15)				
Serum magnesium (mg/dL)	0.55	0.85 (0.50–1.45)				
Serum phosphate (mg/dL)	0.12	1.18 (0.96–1.46)				
Serum creatinine (mg/dL)	0.79	0.96 (0.73–1.28)				
Serum urea (mg/dL)	0.70	1.00 (1.00–1.01)				
Serum chloride (mEq/L)	0.24	0.97 (0.92–1.02)				
Vasopressor dose						
Norepinephrine (μg/min)	0.49	1.05 (0.91–1.21)				
Vasopressin (units/min)	0.30	0.80 (0.52–1.23)				
Biomarkers						
Urine neutrophil gelatinase lipocalin (ng/mL)	0.98	1.00 (0.79–1.27)				
Serum cystatin C (mg/L)	0.17	1.44 (0.86–2.42)				
NT‐pro‐BNP (pg/mL)	0.78	0.17 (0.38–3.67)				
Renal recovery	0.009	0.36 (0.17–0.78)	0.035	0.42 (0.19–0.94)	0.060	0.42 (0.17–1.04)
Shock reversal	0.27	0.67 (0.33–1.37)				
Intradialytic hypotension	0.03	2.25 (1.10–4.59)	0.467	1.34 (0.61–2.95)	0.508	1.34 (0.56–3.18)
Early SLED (EG) vs. Late SLED	0.058	0.51 (0.26–1.02)			0.996	1.00 (0.41–2.45)

*Note:* Data presented as hazard ratio (HR) and 95% CI (confidence intervals) derived from Cox‐regression analysis.

Abbreviations: FiO_2_, fraction of inspired oxygen; IVC, inferior vena cava diameter; KDIGO, kidney disease improving global outcome criteria; PCO_2_, partial pressure of carbon dioxide; PO_2_, partial pressure of oxygen; SOFA, sequential organ failure assessment.

### 4.6 Predictors of Early Deaths at Day 7

Because the deaths at 28 days could be due to various factors attributed to liver disease itself, we performed an additional analysis to understand the predictors associated with early deaths, particularly to assess the complications associated directly with the dialysis strategy. In the first model, ED was independently associated with lower mortality at 7 days (HR 0.34, 0.12–0.95), while in model 2, higher CysC (HR 5.77, 2.49–13.33) and the development of IDH (HR 9.97, 2.28–34.76) were independent predictors of 7‐day mortality (Table S4, Figure 3C,D). The same factors were found significant in the PP analysis (Table S5, Figure 3E,F).

## 5 Discussion

The results of the open label randomized controlled trial performed in patients with cirrhosis and septic shock and advanced renal impairment requiring dialysis show the benefits of a timely initiation of dialysis as a better strategy compared to dialyzing and waiting until the patient meets the absolute criteria. The dilemma of the exact timing of dialysis in patients with cirrhosis and septic shock with severe renal dysfunction is a vexing challenge for the managing clinicians. We observed that waiting until the patient met absolute criteria is associated with a higher incidence of IDH, hemodynamic instability, and a higher need for CRRT. This in turn was associated with a decrease in the incidence of renal function recovery and reversal of shock, resulting in significantly higher early deaths. Renal recovery was associated with improved outcomes. We found that higher levels of CysC predict both renal recovery and mortality. Urine NGAL could predict reversal of shock.

The debate on timing, modality, and dose of dialysis has been ongoing, and still, there is a lack of consensus [6, 7, 8]. Most studies exploring the dialysis strategy have excluded patients with decompensated cirrhosis. In the artificial kidney initiation in kidney injury (AKIKI) trial the early strategy, defined as dialysis initiation within 6 h of meeting KDIGO Stage 3, was comparable to a delayed strategy [8]. The post hoc analysis in the subset of patients with severe sepsis and acute respiratory distress syndrome also failed to show the benefits of early initiation [11]. However, the type of dialysis modality was decided by the attending clinician, and the population of patients included in the trial was very heterogeneous. Contrary to this, in our study, we had a homogeneous cohort of patients with cirrhosis, all with septic shock managed at a single center, and we protocolized a uniform method of dialysis modality in all included patients. SLED is a hybrid modality of dialysis that is currently used as an effective alternative to CRRT in resource‐constrained countries [12, 13]. Our results are consistent with the ELAIN trial, wherein the authors showed the impact of early strategy in improving both short‐and long‐term recovery of renal functions [14].

The development of IDH was significantly higher in the late group compared to the early group. Decline in blood volume and loss of vascular resistance are implicated in the development of this dreaded complication [15, 16]. In patients with cirrhosis, a low systemic vascular resistance and vasodilated state, the burden of inflammatory mediators that accumulate in sepsis and impaired preload could be the most important factor causing IDH. The development of hypotension worsens the renal ischemia and reduces the chances of renal recovery [17, 18]. We identified IDH as an independent predictor of early deaths at Day 7 and lower renal recovery. It was interesting to observe that some degree of hemodynamic instability requiring an increase in the vasopressors was not different between the groups. Patients in the early group had a more effective dialysis as was seen in the degree of urea reduction, tolerance of ultrafiltration (wherever it was required) and also lesser conversion to CRRT. The severity of renal dysfunction, development of IDH, and how the renal failure was managed were independent predictors of early deaths. Patients with cirrhosis could succumb to second infections and many other complications which may have accounted for a nonsignificant difference in survival benefit with early dialysis at Day 28.

Renal recovery from dialysis is a rare phenomenon, and large observational studies have suggested rates varying from 1% to 2.4% [17]. This is because most of these patients either succumb to the severity of the underlying disease or to the lethal extra‐renal complications implicated in ATN [18]. In a large prospective study, we had shown that the organ crosstalk and renal recovery at day seven after therapeutic interventions was an independent predictor of improved outcomes. In our analysis, renal recovery was independently associated with improved survival. Timely dialysis, lack of IDH, lower CysC, reversal of shock, and lactate clearance were independent factors associated with renal recovery. Timely dialysis also averted the metabolic complications, led to better volume control, as was seen with lower need for ultrafiltration, and enabled early hemodynamic optimization in the early compared to the late group [17, 18]. In the early group, possibly the severity and duration of AKI were reduced by a timely dialysis. Evaluation of biomarkers showed higher CysC to be associated with decreased renal recovery. We have previously reported the utility of CysC in predicting chronic kidney disease and AKI in patients with decompensated cirrhosis [19, 20]. We found a cut‐off of less than or equal to 2.75 mg/L CysC as a predictor of renal recovery, and above 3.31 mg/L correlated with early deaths in cirrhotics with septic shock. A lower CysC predicting renal recovery could also indirectly signify the benefits of dialyzing early before significant decline in glomerular functions. We observed very high levels of NGAL in the enrolled patients; however, it could not determine recovery. Immune cells are also a crucial nonrenal source of NGAL in states of systemic inflammation. In these patients, high urine levels could be attributed to increased filtration through the glomerulus by an increased release by the immune cells or decreased absorption in the proximal tubules [19, 20, 21]. In our study, it was intriguing to observe NGAL predicting reversal of shock but not renal recovery or mortality. This is possibly because NGAL is an established marker of renal injury but not repair. In the ELAIN study, urine NGAL was used as a biomarker to identify dialysis initiation in patients [14]. Going by the levels of NGAL, ideally, most of our patients should have undergone timely dialysis. Platelets were identified as an independent predictor of renal recovery. Platelets appear at the crossroad of systemic inflammation, endothelial dysfunction, and coagulation in patients with sepsis‐induced organ dysfunction [22]. Thrombocytopenia is a consequence of platelet activation and consumption and has negative prognostic implications in sepsis [23]. In AKI, platelets worsen renal hypoxia by impairing the hemodynamic responses during AKI. Future prospective studies are needed to explore the cross‐talk of platelets in inflammation‐driven AKI and other mediators as biomarkers of AKI recovery.

The current trial was performed in an extremely difficult‐to‐treat group of patients with cirrhosis with septic shock and renal failure requiring dialysis. In addition, we have shown the utility of renal biomarkers, that is, CysC and NGAL in these patients. Further, for the first time, we showed the safety and efficacy of performing an appropriately timed SLED in these patients. Our study also brings out the significance of managing kidneys in patients with cirrhosis with septic shock and the impact of renal recovery which directly influenced patient outcomes. However, our study also has several limitations. The first and foremost being a single‐center design and therefore, the results need to be externally validated. The second was the small sample size. We performed an interim analysis, which had shown many patients developing IDH and conversion to CRRT in the late group, based on which the trial protocol was revised. Third, the estimation of the effect size is most likely to be vastly different from the effect size in the population. Fourth, the post hoc analysis was not prespecified in the initial protocol.

Considering the sick population who succumb to other complications, we could not demonstrate the benefits of timing the dialysis on 28‐day survival. It was therefore decided to terminate the trial after enrolling 50 patients. Also, many patients chose end‐of‐life care and de‐escalation of medical support, considering futility. Further, none of the patients underwent liver transplant despite initial recovery from shock within 28 days, which is a limitation.

To summarize, the results of the current open‐label randomized controlled trial in patients with cirrhosis and septic shock requiring dialysis showed the benefits of an early initiation of dialysis in improving renal outcomes. In the context of septic shock, waiting for absolute criteria is fraught with more hemodynamic instability, higher renal ischemia, and ineffective management of renal failure, causing worse outcomes that directly impact mortality. Despite the benefits seen, this study cannot clearly recommend for or against early dialysis due to the small sample size and differences in the groups at the outset. Future studies should explore the role of renal biomarkers along with clinical criteria for decision‐making for dialysis initiation in these patients.

## Disclosure

The authors have nothing to report.

## Consent

It should be patient’s next of kin or legal representative.

## Conflicts of Interest

The authors declare no conflicts of interest.

## Supporting information


**Data S1.**Supporting Information.

## Data Availability

Data will be available in excel format on request.
