# Supplementary material for: Early Versus Late Dialysis in Cirrhosis Patients and Septic Shock (ELDICS Study): A Randomized Controlled Trial (NCT02937961)
Source: JGH Open. 2025 Sep 24;9(9):e70216. doi: 10.1002/jgh3.70216 (PMC12460919; doi:10.1002/jgh3.70216)
Supplement: Supplementary file 1 — Data S1.Supporting Information. [file JGH3-9-e70216-s001.docx]

**Supplementary Appendix**

This appendix has been provided by the authors to give readers additional information about their work.

Supplement to: Maiwall R et al.

Early versus late dialysis in cirrhosis with septic shock and acute kidney injury- [The ELDICS Randomized Controlled Trial]

Rakhi Maiwall^1^, Samba Siva Rao Pasulapeti^2^, Prashant Agarwal^3^, Sherin Thomas^4^, Harsh Vardhan Tevethia^1^, Rajendra Prasad Mathur^5^, Shiv Kumar Sarin^1^

**Table of contents**

| Supplementary Methods | Page 2,3 |
| --- | --- |
| Definitions | Page 4,6 |
| References | Page 7 |
| Supple Tables | Page 8-15 |

**Summary of the Protocol for Patient Management**

Patients were managed according to the standard of care. At admission, all patients underwent a thorough physical examination and baseline investigations. Pulse rate, systolic and diastolic blood pressure and mean arterial blood pressure were monitored dynamically in all patients. Urinary catheterization was performed in parallel and which was then serially assessed hourly during the ICU stay. Blood for investigations was collected for each patient and sent for investigations within 30 minutes of patient enrollment. Patients requiring mechanical ventilation underwent the same either in the emergency department or on arrival in the intensive care unit. A record of central venous pressure (CVP) or inferior vena cava diameter were performed for guidance for fluid. The resuscitation fluid was according to the dynamic indices which included 5% albumin with or without crystalloids. Patients, who did not respond to fluid replacement at 2-3 h received were initiated on vasopressors. Norepinephrine was the first-choice vasopressor in all patients. It was initiated at a dose of 7.5 μg/min rate and increased to a maximum of 60 μg/min. In patients who failed to achieve a mean arterial pressure with 0.5ug/kg/min of norepinephrine, initiation of second vasopressors included either terlipressin or vasopressin along with intravenous low dose hydrocortisone. Broad spectrum antibiotics were instituted in accordance with the Institute’s protocol. The antibiotics were revised based on cultures. Patients were initiated on early enteral nutrition. The patients were monitored in the intensive care unit with hourly monitoring of mean arterial pressure, urine output and measurement of arterial lactate every 4-6 hours. Regular microbiologic screening for infection in blood, urine, ascitic fluid, sputum or mini- bronchoalveolar lavage (mini-BAL) fluid for patients on mechanical ventilation support every 48-72 hours along with daily chest X-Ray were done for each patient. Daily MELD, CTP and SOFA scores were calculated to prognosticate the patients.

**PROTOCOL OF DIALYSIS**

SLED sessions were targeted to 8-10 hours in duration with blood and dialysate flows of 150-200 mL/min and 300ml/min, respectively. SLED was delivered using the dialysis machines (Fresenius, 4008S, Dialyser F6 HPS). The minimum frequency of SLED treatments was three times per week. In patients who developed hemodynamic instability, continuous renal replacement therapy was administered as the rescue strategy as continuous venovenous hemodiafiltration (CVVHDF) using Prisma and Prismaflex (Gambro) devices, with blood flows ranging from 150–180 mL/hr and target effluent rates of 20 – 25 mL/kg/hr. Anticoagulation was not used during dialysis. The dialysis sessions were continued until renal recovery, discharge, or death. The dialysis sessions were also discontinued in patients who opted for end -of life care considering futility of intensive care.

**Definitions**

- Septic Shock: Clinical construct of sepsis with persisting hypotension requiring vasopressors to maintain MAP>=65 mm of Hg with serum lactate >2 mmol/L despite adequate volume resuscitation^1^
- Intradialytic Hypotension: Decrease in systolic blood pressure by ≥20 mm Hg or a decrease in MAP by 10 mm Hg associated with symptoms^2,3^
- Hemodynamic stability: No increase in vasopressor dose with dialysis
- Renal Recovery: Discontinuation of maintenance dialysis with increase in urine output to more than 400 ml/day in patients who were anuric ^2,3^
- Achievement of Target Ultrafiltration: Ultrafiltration planned before initiation of dialysis for patients with volume overload
- Lactate clearance: lactate baseline - lactate at time point/baseline lactate x100^3^
- Cirrhosis-The diagnosis of cirrhosis was considered based on clinical, biochemical, imaging (ultrasonography, computed tomography or magnetic resonance imaging), and endoscopic ﬁndings or liver biopsy (when available)^5^.

Bacterial infections were diagnosed according to the following criteria:

- Spontaneous bacterial peritonitis (SBP): ascitic fluid polymorphonuclear cell count ≥ 250/mm^3 5^
- Urinary tract infections (UTI): Patient with a positive urine culture or with signs or symptoms (fever ≥ 38° C, urgency, frequency, dysuria, or suprapubic tenderness) and more than 10 leukocytes/µL in urine^6^.
- Pneumonia: radiologic evidence of a new, or progression of a previous, pulmonary infiltrate, consolidation or cavitation plus at least one of the following symptoms (new onset of purulent sputum or change in character of sputum, new onset of cough, dyspnea or tachypnea >20 breaths per minute, rales or bronchial breath sounds or worsening of gas exchange) and/or organisms cultured from blood, pleural fluid or a specimen obtained by transtracheal, aspirate, bronchoalveolar lavage, or biopsy
- Spontaneous bacteremia: Positive blood culture (at least two positive blood cultures for common skin contaminant), in the absence of a known source of infection
- Other infections were diagnosed according to the Centers for Disease Control and Prevention Criteria^6^

**Per-protocol analysis**

On per-protocol analysis, the number of deaths were higher in the LG compared to EG, (76% vs. 56%; p= 0.14) but the difference was not statistically significant [**Figure 2C]**. Early deaths (i.e., deaths within the first 7 days of admission) were significantly higher in the LG compared to EG (56% [10 out of 18] vs 21% [5 out of 24]; p=0.027). [**Figure 2D**] Of these deaths, 53% (8 out of 15) patients had developed intra-dialytic hypotension during dialysis. Majority of these patients were in the late group (70% [7 out of 10] vs 20% [1 out of 5]). The overall incidence of intradialytic hypotension was significantly higher in LG compared to EG (50% vs. 13%; p=0.008), hemodynamic stability without increase in the vasopressors was better in EG (56% vs. 75%; p=0.19), with higher urea reduction ratio (74% vs. 47%; p=0.09), achievement of target ultrafiltration goals (64% vs. 42%; p=0.22) compared to LG respectively. The duration of mechanical ventilation (in days) (4.08±3.28 vs. 5.39±4.67; p=0.29), intensive care unit stay (5.74±3.33 vs. 6.33±4.69; p=0.64). The incidence of renal recovery (71% vs. 11%; p<0.001) and reversal of shock (58% vs. 17%; p=0.006) was significantly higher in the EG compared to LG respectively. [**Table 2**]

**REFERENCES**

- 1. Briegel J, Möhnle P. Internationale Leitlinien der Surviving Sepsis Campaign : Update 2016 [International guidelines from the Surviving Sepsis Campaign : 2016 update. Anaesthesist. 2017;66:530-538.
  2. Chawla LS, Bellomo R, Bihorac A, Goldstein SL, Siew ED, Bagshaw SM; Acute Disease Quality Initiative Workgroup 16.. Acute kidney disease and renal recovery: consensus report of the Acute Disease Quality Initiative (ADQI) 16 Workgroup. Nat Rev Nephrol. 2017;13:241-257.
  3. Ghaffar U, Easom AK. A quality improvement project: Strategies to reduce intradialytic hypotension in hemodialysis patients. Nephrol News Issues. 2015;29: 30, 32, 34 passim
  4. Hernandez G, Bellomo R, Bakker J. The ten pitfalls of lactate clearance in sepsis. Intensive Care Med. 2019; 45:82-85.
  5. Rimola A, García-Tsao G, Navasa M, et al. Diagnosis, treatment and prophylaxis of spontaneous bacterial peritonitis: a consensus document. International Ascites Club. *J Hepatol*. 2000;32.
  6. Horan TC, Andrus M, Dudeck MA. CDC/NHSN surveillance definition of health care-associated infection and criteria for specific types of infections in the acute care setting. *Am J Infect Control*. 2008;36:309-332.

**Supple Table 1: Intention-to-treat and per-protocol analysis between the two groups stratified by intervention.**

|  | Intention-to-treat analysis (ITT) | | | Per-protocol analysis (PP) | | |
| --- | --- | --- | --- | --- | --- | --- |
| **Primary Outcome** | Early group  (n=25) | Late group  (n=25) | P value | Early group  (n=24) | Late group  (n=18) | P value |
| 28-day mortality | 14 (56) 19 | 19 (76) | 0.14 | 1 (4) | 3 (17) | 0.30 |
| **Secondary Outcome** |  |  |  |  |  |  |
| Incidence of intradialytic hypotension | 3 (12) | 12 (48) | 0.005 | 3 (13) | 9 (50) | 0.008 |
| Hemodynamic stability (no increase in vasopressors) | 19 (76) | 14 (56) | 0.14 | 18 (75) | 10 (56) | 0.19 |
| Urea reduction ratio | 18 (75) | 9 (41) | 0.019 | 17 (74) | 7 (47) | 0.09 |
| Achievement of target ultrafiltration | 15(65.2) | 6 (33) | 0.043 | 14 (64) | 5 (42) | 0.22 |
| Renal recovery | 17 (68) | 3 (12) | <0.001 | 17 (71) | 2 (11) | <0.001 |
| Reversal of shock | 15 (60) | 4 (16) | 0.001 | 14 (58) | 3 (17) | 0.006 |
| Days of mechanical ventilation | 3.92±  3.32 | 5.56± 4.39 | 0.14 | 4.08 ±3.28 | 5.39 ±4.67 | 0.29 |
| Days of intensive care unit | 5.67  ±3.28 | 6.28 ±4.52 | 0.59 | 5.74 ±3.33 | 6.33 ±4.69 | 0.64 |
| 28-day mortality | 14 (56) | 19 (76) | 0.14 | 13 (54) | 12 (67) | 0.41 |

Data presented as Chi-square test for categorical variables and independent student’s T-test for continuous variables. The p value for 28-day mortality is derived from Cox-regression analysis. Data has been presented as number (percentage) for categorical variables and mean±standard deviation for continuous variables.

**Supplementary Table 2: Predictors of renal recovery-Logistic regression analysis (Per-protocol analysis)**

|  |  | | | **Model 1** | | | **Model 2** | | |
| --- | --- | --- | --- | --- | --- | --- | --- | --- | --- |
|  | **P-value** | **Unadjusted OR** | **95% CI for OR** | **P-value** | **Adjusted OR** | **95% CI for OR** | **P-value** | **Adjusted OR** | **95% CI for OR** |
| Age (in years) | 0.952 | 1.00 | 0.94-1.07 |  |  |  |  |  |  |
| Gender (Males); n (%) | 0.238 | 0.24 | 0.02-2.55 |  |  |  |  |  |  |
| Etiology (Alcohol); n (%) | 0.612 | 1.39 | 0.39-5.01 |  |  |  |  |  |  |
| **Severity scores** |  |  |  |  |  |  |  |  |  |
| Model for end-stage liver disease | 0.846 | 0.99 | 0.90-1.09 |  |  |  |  |  |  |
| Sequential organ failure assessment | 0.902 | 1.01 | 0.84-1.22 |  |  |  |  |  |  |
| Child turcott pugh score | 0.889 | 0.97 | 0.65-1.46 |  |  |  |  |  |  |
| **Physiological parameters** |  |  |  |  |  |  |  |  |  |
| Mean Arterial Pressure (mm of Hg) | 0.525 | 0.98 | 0.94-1.03 |  |  |  |  |  |  |
| Heart rate (beats/min) | 0.753 | 0.99 | 0.96-1.03 |  |  |  |  |  |  |
| Respiratory rate (/min) | 0.898 | 0.99 | 0.82-1.19 |  |  |  |  |  |  |
| Urine Output(ml/hour) | 0.543 | 1.02 | 0.97-1.07 |  |  |  |  |  |  |
| KDIGO; n (%)  Stage 3 vs.stage 2 | 0.276 | 0.49 | 0.13-1.78 |  |  |  |  |  |  |
| FiO_2_ (%) | 0.909 | 1.00 | 0.97-1.02 |  |  |  |  |  |  |
| Arterial lactate (mmol/L) | 0.132 | 0.76 | 0.53-1.09 |  |  |  |  |  |  |
| Central Venous Pressure  (in cms) | 0.466 | 1.10 | 0.85-1.43 |  |  |  |  |  |  |
| IVC (diameter in cm) | 0.906 | 0.99 | 0.82-1.20 |  |  |  |  |  |  |
| pH |  |  |  |  |  |  |  |  |  |
| pCO_2_ (mm of Hg) | 0.532 | 0.98 | 0.94-1.03 |  |  |  |  |  |  |
| pO_2_ (mm of Hg) | 0.380 | 0.99 | 0.98-1.01 |  |  |  |  |  |  |
| **Biochemical parameters** |  |  |  |  |  |  |  |  |  |
| Total leucocyte count (x10^3^ cells/mm^3^) | 0.268 | 1.05 | 0.97-1.13 |  |  |  |  |  |  |
| Platelet count (x10^3^ cells/mm^3^) | 0.011 | 1.01^*^ | 1.00-1.03 | 0.021 | 1.02^*^ | 1.00-1.04 | 0.032 | 1.02^*^ | 1.00-1.04 |
| Serum total bilirubin (mg/dl) | 0.504 | 0.98 | 0.91-1.05 |  |  |  |  |  |  |
| International normalized ratio | 0.704 | 0.87 | 0.42-1.80 |  |  |  |  |  |  |
| Hemoglobin (g/dl) | 0.282 | 0.85 | 0.63-1.14 |  |  |  |  |  |  |
| Serum sodium (mEq/L) | 0.498 | 0.97 | 0.90-1.05 |  |  |  |  |  |  |
| Serum potassium (mEq/L) | 0.354 | 1.55 | 0.61-3.90 |  |  |  |  |  |  |
| Serum bicarbonate (mEq/L) | 0.684 | 1.03 | 0.89-1.19 |  |  |  |  |  |  |
| Anion gap | 0.664 | 1.02 | 0.93-1.13 |  |  |  |  |  |  |
| Serum calcium (mg/dl) | 0.030 | 1.23^*^ | 1.02-1.47 |  |  |  |  |  |  |
| Serum magnesium (mg/dl) | 0.125 | 3.16 | 0.73-13.72 |  |  |  |  |  |  |
| Serum phosphate (mg/dl) | 0.163 | 0.70 | 0.43-1.15 |  |  |  |  |  |  |
| Serum creatinine (mg/dl) | 0.708 | 0.89 | 0.48-1.66 |  |  |  |  |  |  |
| Serum urea (mg/dl) | 0.818 | 1.00 | 0.99-1.01 |  |  |  |  |  |  |
| Serum chloride (mEq/L) | 0.044 | 1.15^*^ | 1.00-1.31 |  |  |  |  |  |  |
| **Vasopressor dose** |  |  |  |  |  |  |  |  |  |
| Norepinephrine | 0.547 | 1.08 | 0.84-1.39 |  |  |  |  |  |  |
| Vasopressin | 0.865 | 0.95 | 0.51-1.75 |  |  |  |  |  |  |
| **Biomarkers** |  |  |  |  |  |  |  |  |  |
| Urine neutrophil gelatinase lipocalin (ng/ml) | 0.403 | 0.83 | 0.54-1.28 |  |  |  |  |  |  |
| Serum cystatin c (mg/L) | 0.019 | 0.26^*^ | 0.09-0.81 | 0.085 | 0.15 | 0.02-1.30 | 0.017 | 0.06^*^ | 0.01-0.60 |
| NT-pro-BNP | 0.394 | 0.55 | 0.14-2.18 |  |  |  |  |  |  |
| Shock reversal | 0.009 | 6.17^**^ | 1.58-24.05 | 0.191 | 4.09 | 0.50-33.71 | 0.537 | 1.99 | 0.22-17.72 |
| Early dialysis | 0.001 | 19.43^**^ | 3.50-107.78 | 0.030 | 16.68^*^ | 1.32-211.10 |  |  |  |
| Intradialytic hypotension | 0.029 | 0.15^*^ | 0.03-0.82 |  |  |  | 0.099 | 0.08 | 0.00-1.59 |
| Lactate clearance | 0.042 | 4.00^*^ | 1.05-15.26 | 0.395 | 3.30 | 0.21-51.69 | 0.156 | 5.94 | 0.51-69.75 |

Abbreviations: SOFA sequential organ failure assessment, FiO_2_ fraction of inspired oxygen, PO_2_ partial pressure of oxygen, and PCO_2_ partial pressure of carbon dioxide.IVC- inferior vena cava diameter, KDIGO-kidney disease improving global outcome criteria

Data presented as odd’s ratio (OR) and 95% CI (confidence intervals) derived from binary logistic regression analysis

**Supplementary Table 3 : Predictors of 28-day mortality-Cox-regression analysis (per-protocol analysis)**

|  |  | **Model 1** |  |  | **Model 2** |  |  | **Model 3** |  |
| --- | --- | --- | --- | --- | --- | --- | --- | --- | --- |
|  | **p-value** | **Unadjusted HR** | **95% CI** | **p-value** | **Adjusted HR** | **95% CI** | **p-value** | **Adjusted HR** | **95% CI** |
| Age (in years) | 0.975 | 1.00 | 0.962-1.038 |  |  |  |  |  |  |
| Gender (Males); n (%) | 0.239 | 3.33 | (0.45-24.66) |  |  |  |  |  |  |
| Etiology (Alcohol); n (%) | 0.948 | 1.03 | 0.453-2.330 |  |  |  |  |  |  |
| **Severity scores** |  |  |  |  |  |  |  |  |  |
| Model for end-stage liver disease | 0.800 | 1.01 | 0.948-1.071 |  |  |  |  |  |  |
| Sequential organ failure assessment | 0.021 | 1.18^*^ | 1.025-1.351 | 0.075 | 1.14 | 0.987-1.318 | 0.085 | 1.14 | 0.982-1.317 |
| Child turcott pugh score | 0.946 | 1.01 | 0.784-1.299 |  |  |  |  |  |  |
| **Physiological parameters** |  |  |  |  |  |  |  |  |  |
| Mean Arterial Pressure (mm of Hg) | 0.782 | 1.00 | 0.964-1.028 |  |  |  |  |  |  |
| Urine Output(ml/hour) | 0.646 | 1.01 | 0.976-1.040 |  |  |  |  |  |  |
| KDIGO; n (%)  Stage 3 vs. Stage 2 | 0.393 | 1.46 | 0.611-3.506 |  |  |  |  |  |  |
| FiO_2_ (%) | 0.675 | 1.00 | 0.989-1.018 |  |  |  |  |  |  |
| Arterial lactate (mmol/L) | 0.018 | 1.23^*^ | 1.037-1.470 | 0.047 | 1.22* | 1.002-1.479 | 0.043 | 1.23* | 1.006-1.504 |
| Central Venous Pressure  (in cms) | 0.904 | 1.01 | 0.857-1.190 |  |  |  |  |  |  |
| IVC (diameter in cm) | 0.480 | 1.05 | 0.923-1.185 |  |  |  |  |  |  |
| pH | 0.577 | 0.36 | 0.009-13.440 |  |  |  |  |  |  |
| pCO_2_ (mm of Hg) | 0.659 | 1.01 | 0.980-1.033 |  |  |  |  |  |  |
| pO_2_ (mm of Hg) | 0.340 | 0.99 | 0.984-1.006 |  |  |  |  |  |  |
| **Biochemical parameters** |  |  |  |  |  |  |  |  |  |
| Total leucocyte count (x10^3^ cells/mm^3^) | 0.411 | 1.02 | 0.974-1.066 |  |  |  |  |  |  |
| Platelet count (x10^3^ cells/mm^3^) | 0.164 | 0.65 | 0.350-1.195 |  |  |  |  |  |  |
| Serum total bilirubin (mg/dl) | 0.995 | 1.00 | 0.909-1.099 |  |  |  |  |  |  |
| International normalized ratio | 0.297 | 1.03 | 0.970-1.104 |  |  |  |  |  |  |
| Hemoglobin (g/dl) | 0.825 | 1.01 | 0.906-1.132 |  |  |  |  |  |  |
| Serum sodium (mEq/L) | 0.519 | 0.81 | 0.437-1.518 |  |  |  |  |  |  |
| Serum potassium (mEq/L) | 0.072 | 1.25 | 0.980-1.591 |  |  |  |  |  |  |
| Serum bicarbonate (mEq/L) | 0.516 | 1.05 | 0.900-1.235 |  |  |  |  |  |  |
| Anion gap | 0.592 | 0.88 | 0.561-1.390 |  |  |  |  |  |  |
| Serum calcium (mg/dl) | 0.789 | 0.95 | 0.651-1.386 |  |  |  |  |  |  |
| Serum magnesium (mg/dl) | 0.974 | 1.00 | 0.993-1.007 |  |  |  |  |  |  |
| Serum phosphate | 0.199 | 0.96 | 0.891-1.024 |  |  |  |  |  |  |
| Serum creatinine (mg/dl) | 0.743 | 1.23 | 0.361-4.168 |  |  |  |  |  |  |
| Serum urea (mg/dl) | . | 1.00 | 1.000-1.000 |  |  |  |  |  |  |
| Serum chloride (mEq/L) | 0.019 | 0.36^*^ | 0.156-0.844 |  |  |  |  |  |  |
| **Vasopressor dose** |  |  |  |  |  |  |  |  |  |
| Norepinephrine | 0.861 | 0.98 | 0.753-1.267 |  |  |  |  |  |  |
| Vasopressin | 0.226 | 1.44 | 0.797-2.612 |  |  |  |  |  |  |
| Shock reversal | 0.525 | 0.77 | 0.346-1.719 |  |  |  | 0.050 | 0.39^*^ | 0.154-0.999 |
| Renal recovery | 0.019 | 0.36^*^ | 0.156-0.844 | 0.05 | 0.39* | 0.154-0.999 | 0.025 | 0.30* | 0.104-0.862 |
| Intradialytic hypotension | 0.042 | 2.35^*^ | 1.032-5.335 |  |  |  | 0.297 | 1.74 | 0.615-4.901 |
| Early SLED vs. Late SLED | 0.228 | 0.62 | 0.280-1.354 |  |  |  | 0.348 | 1.67 | 0.573-4.875 |

Abbreviations: SOFA sequential organ failure assessment, FiO_2_ fraction of inspired oxygen, PO_2_ partial pressure of oxygen, and PCO_2_ partial pressure of carbon dioxide.IVC- inferior vena cava diameter, KDIGO-kidney disease improving global outcome criteria

Data presented as hazard ratio (HR) and 95% CI (confidence intervals) derived from Cox-regression analysis

**Supple Table 4: Predictors of 7-day mortality-Cox-regression analysis (intention-to-treat- n=50)**

|  |  | | | **Model 1** | | | **Model 2** | | |  |
| --- | --- | --- | --- | --- | --- | --- | --- | --- | --- | --- |
| **Variables** | **p-value** | **Unadjusted HR** | **95% CI** | **p-value** | **Adjusted HR** | **95% CI** | | **p-value** | **Adjusted HR** | **95% CI** |
| Age (in years) | 0.358 | 0.98 | 0.93-1.03 |  |  |  | |  |  |  |
| ##Gender (Males); n (%) | 1 | 1.78E+15 | 0.00- |  |  |  | |  |  |  |
| Etiology (Alcohol); n (%) | 0.581 | 1.32 | 0.49-3.51 |  |  |  | |  |  |  |
| **Severity scores** |  |  |  |  |  |  | |  |  |  |
| Model for end-stage liver disease | 0.849 | 0.99 | 0.93-1.06 |  |  |  | |  |  |  |
| Sequential organ failure assessment | 0.04 | 1.18* | 1.01-1.38 | 0.068 | 1.15 | 0.99-1.34 | | 0.052 | 1.16 | 1.00-1.35 |
| Child turcott pugh score | 0.899 | 1.02 | 0.72-1.45 |  |  |  | |  |  |  |
| **Physiological parameters** |  |  |  |  |  |  | |  |  |  |
| Mean Arterial Pressure (mm of Hg) | 0.561 | 0.99 | 0.96-1.02 |  |  |  | |  |  |  |
| Urine Output(ml/hour) | 0.214 | 1.02 | 0.99-1.04 |  |  |  | |  |  |  |
| KDIGO; n (%)  Stage 3 vs. stage 2 | 0.428 | 1.52 | 0.54-4.26 |  |  |  | |  |  |  |
| FiO_2_ (%) | 0.495 | 1.01 | 0.99-1.02 |  |  |  | |  |  |  |
| Arterial lactate (mmol/L) |  |  |  |  |  |  | |  |  |  |
| IVC (diameter in cm) | 0.458 | 0.95 | 0.83-1.09 |  |  |  | |  |  |  |
| Central venous pressure (in cm) |  |  |  |  |  |  | |  |  |  |
| pH | 0.651 | 0.36 | 0.00-29.38 |  |  |  | |  |  |  |
| pCO_2_ (mm of Hg) | 0.779 | 1.00 | 0.98-1.03 |  |  |  | |  |  |  |
| pO_2_ (mm of Hg) | 0.395 | 0.99 | 0.98-1.01 |  |  |  | |  |  |  |
| **Biochemical parameters** |  |  |  |  |  |  | |  |  |  |
| Total leucocyte count (x10^3^ cells/mm^3^) | 0.561 | 1.02 | 0.96-1.07 |  |  |  | |  |  |  |
| Platelet count (x10^3^ cells/mm^3^) | 0.722 | 1.00 | 0.99-1.01 |  |  |  | |  |  |  |
| Serum total bilirubin (mg/dl) | 0.151 | 1.03 | 0.99-1.07 |  |  |  | |  |  |  |
| International normalized ratio | 0.83 | 1.06 | 0.65-1.73 |  |  |  | |  |  |  |
| Hemoglobin (g/dl) | 0.861 | 1.02 | 0.82-1.27 |  |  |  | |  |  |  |
| Serum sodium (mEq/L) | 0.96 | 1.00 | 0.95-1.06 |  |  |  | |  |  |  |
| Serum potassium (mEq/L) | 0.303 | 0.7 | 0.35-1.38 |  |  |  | |  |  |  |
| Serum bicarbonate (mEq/L) | 0.281 | 0.94 | 0.84-1.05 |  |  |  | |  |  |  |
| Anion gap | 0.395 | 1.03 | 0.96-1.10 |  |  |  | |  |  |  |
| Serum calcium (mg/dl) | 0.325 | 0.94 | 0.82-1.07 |  |  |  | |  |  |  |
| Serum magnesium (mg/dl) | 0.071 | 0.35 | 0.11-1.09 |  |  |  | |  |  |  |
| Serum phosphate (mg/dl) | 0.642 | 1.07 | 0.80-1.42 |  |  |  | |  |  |  |
| Serum creatinine (mg/dl) | 0.059 | 0.54 | 0.28-1.02 |  |  |  | |  |  |  |
| Serum urea (mg/dl) | 0.944 | 1.00 | 0.99-1.01 |  |  |  | |  |  |  |
| Serum chloride | 0.676 | 0.99 | 0.92-1.06 |  |  |  | |  |  |  |
| **Vasopressor dose** |  |  |  |  |  |  | |  |  |  |
| Norepinephrine | 0.373 | 1.09 | 0.90-1.31 |  |  |  | |  |  |  |
| Vasopressin | 0.812 | 0.93 | 0.53-1.64 |  |  |  | |  |  |  |
| **Biomarkers** |  |  |  |  |  |  | |  |  |  |
| Urine neutrophil gelatinase lipocalin (ng/ml) | 0.432 | 1.17 | 0.79-1.71 |  |  |  | |  |  |  |
| Serum cystatin c (mg/L) | 0.007 | 2.38** | 1.27-4.45 |  |  |  | | <0.001 | 5.77** | 2.49-13.33 |
| NT-pro-BNP | 0.578 | 0.71 | 0.21-2.40 |  |  |  | |  |  |  |
| Renal recovery | 0.009 | 0.14** | 0.03-0.61 |  |  |  | |  |  |  |
| Reversal of shock | 0.092 | 0.38 | 0.13-1.17 |  |  |  | |  |  |  |
| Early dialysis vs. Late | 0.029 | 0.32* | 0.11-0.89 | 0.04 | 0.34* | 0.12-0.95 | |  |  |  |
| Intradialytic hypotension | 0.002 | 4.54** | 1.78-11.57 |  |  |  | | <0.001 | 9.97** | 2.86-34.76 |

Abbreviations: SOFA sequential organ failure assessment, FiO_2_ fraction of inspired oxygen, PO_2_ partial pressure of oxygen, and PCO_2_ partial pressure of carbon dioxide.IVC- inferior vena cava diameter, KDIGO-kidney disease improving global outcome criteria

Data presented as hazard ratio (HR) and 95% CI (confidence intervals) derived from Cox-regression analysis

## Confidence interval cannot be precisely calculated as there were extremely few events (just 2 events) for women patients.

**Supplementary Table 5: Predictors of 7-day mortality-Cox-regression analysis (per-protocol- n=42)**

|  | **Unadjusted** | | | **Model 1** | | | **Model 2** | | |
| --- | --- | --- | --- | --- | --- | --- | --- | --- | --- |
| **Variables** | **p-value** | **Unadjusted HR** | **95% CI** | **p-value** | **Adjusted HR** | **95% CI** | **p-value** | **Adjusted HR** | **95% CI** |
| Age (in years) | 0.751 | 0.99 | 0.942-1.044 |  |  |  |  |  |  |
| ##Gender (Males); n (%) | 1 | 4.21E+15 | 0.000-. |  |  |  |  |  |  |
| Etiology (Alcohol); n (%) | 0.906 | 1.07 | 0.364-3.124 |  |  |  |  |  |  |
| **Severity scores** |  |  |  |  |  |  |  |  |  |
| Model for end-stage liver disease | 0.695 | 0.99 | 0.918-1.058 |  |  |  |  |  |  |
| Sequential organ failure assessment | 0.042 | 1.18* | 1.006-1.388 | 0.061 | 1.15 | 0.994-1.341 | 0.083 | 1.15 | 0.982-1.346 |
| Child turcott pugh score | 0.932 | 1.02 | 0.719-1.433 |  |  |  |  |  |  |
| **Physiological parameters** |  |  |  |  |  |  |  |  |  |
| Mean Arterial Pressure (mm of Hg) | 0.679 | 0.99 | 0.950-1.034 |  |  |  |  |  |  |
| Urine Output(ml/hour) | 0.732 | 1.01 | 0.967-1.049 |  |  |  |  |  |  |
| KDIGO; n (%)  Stage 3 vs. stage 2 | 0.226 | 2.19 | 0.616-7.748 |  |  |  |  |  |  |
| FiO_2_ (%) | 0.453 | 1.01 | 0.989-1.025 |  |  |  |  |  |  |
| Arterial lactate (mmol/L) | 0.064 | 1.24 | 0.987-1.565 |  |  |  |  |  |  |
| Central venous pressure (in cm) | 0.518 | 1.07 | 0.875-1.305 |  |  |  |  |  |  |
| IVC (diameter in cm) | 0.832 | 0.98 | 0.842-1.148 |  |  |  |  |  |  |
| pH | 0.217 | 0.05 | 0.000-5.733 |  |  |  |  |  |  |
| pCO_2_ (mm of Hg) | 0.53 | 1.01 | 0.982-1.036 |  |  |  |  |  |  |
| pO_2_ (mm of Hg) | 0.302 | 0.99 | 0.976-1.008 |  |  |  |  |  |  |
| **Biochemical parameters** |  |  |  |  |  |  |  |  |  |
| Total leucocyte count (x10^3^ cells/mm^3^) | 0.883 | 1 | 0.946-1.067 |  |  |  |  |  |  |
| Platelet count (x10^3^ cells/mm^3^) | 0.759 | 1 | 0.991-1.007 |  |  |  |  |  |  |
| Serum total bilirubin (mg/dl) | 0.096 | 1.05 | 0.992-1.102 |  |  |  |  |  |  |
| International normalized ratio | 0.574 | 0.82 | 0.401-1.659 |  |  |  |  |  |  |
| Hemoglobin (g/dl) | 0.209 | 1.15 | 0.923-1.443 |  |  |  |  |  |  |
| Serum sodium (mEq/L) | 0.906 | 1 | 0.933-1.063 |  |  |  |  |  |  |
| Serum potassium (mEq/L) | 0.146 | 0.55 | 0.244-1.232 |  |  |  |  |  |  |
| Serum bicarbonate (mEq/L) | 0.143 | 0.91 | 0.793-1.034 |  |  |  |  |  |  |
| Anion gap | 0.489 | 1.03 | 0.953-1.107 |  |  |  |  |  |  |
| Serum calcium (mg/dl) | 0.3 | 0.92 | 0.798-1.072 |  |  |  |  |  |  |
| Serum magnesium (mg/dl) | 0.143 | 0.38 | 0.107-1.382 |  |  |  |  |  |  |
| Serum phosphate (mg/dl) | 0.328 | 1.16 | 0.865-1.542 |  |  |  |  |  |  |
| Serum creatinine (mg/dl) | 0.136 | 0.59 | 0.291-1.183 |  |  |  |  |  |  |
| Serum urea (mg/dl) | 0.692 | 1 | 0.993-1.010 |  |  |  |  |  |  |
| Serum chloride (mEq/L) | 0.11 | 0.93 | 0.847-1.017 |  |  |  |  |  |  |
| **Vasopressor dose** |  |  |  |  |  |  |  |  |  |
| Norepinephrine | 0.456 | 1.08 | 0.884-1.315 |  |  |  |  |  |  |
| Vasopressin | 0.926 | 0.97 | 0.548-1.729 |  |  |  |  |  |  |
| **Biomarkers** |  |  |  |  |  |  |  |  |  |
| Urine neutrophil gelatinase lipocalin (ng/ml) | 0.451 | 1.17 | 0.782-1.741 |  |  |  |  |  |  |
| Serum cystatin c (mg/L) | 0.044 | 2.05* | 1.020-4.137 |  |  |  | 0 | 4.88** | 2.024-11.747 |
| NT-pro-BNP | 0.644 | 0.73 | 0.198-2.728 |  |  |  |  |  |  |
| Renal recovery | 0.009 | 0.14** | 0.031-0.613 |  |  |  |  |  |  |
| Reversal of shock | 0.18 | 0.46 | 0.145-1.436 |  |  |  |  |  |  |
| Intradialytic hypotension | 0.005 | 4.28** | 1.546-11.871 |  |  |  | 0.001 | 9.61** | 2.442-37.803 |
| Early dialysis vs. Late | 0.034 | 0.31* | 0.107-0.915 | 0.041 | 0.32* | 0.109-0.954 |  |  |  |

Abbreviations: SOFA sequential organ failure assessment, FiO_2_ fraction of inspired oxygen, PO_2_ partial pressure of oxygen, and PCO_2_ partial pressure of carbon dioxide.IVC- inferior vena cava diameter, KDIGO-kidney disease improving global outcome criteria

Data presented as hazard ratio (HR) and 95% CI (confidence intervals) derived from Cox-regression analysis

## Confidence interval cannot be precisely calculated as there were extremely few events (just 2 events) for women patients.
